# Breastfeeding by a mother taking cyclosporine for nephrotic syndrome

**DOI:** 10.1186/s13006-022-00514-4

**Published:** 2022-10-17

**Authors:** Ruizhe Li, Chuan Zhang, Hongjing Wang, Yunfei An

**Affiliations:** 1 Department of Obstetrics and Gynecology, West China Second University Hospital of Sichuan University, Chengdu, China; 2 Key Laboratory of Birth Defects and Related Diseases of Women and Children (Sichuan University), Ministry of Education, Chengdu, China; 3 Department of Pharmacology, West China Second University Hospital of Sichuan University, Chengdu, China; 4 Department of Laboratory Medicine, West China Hospital of Sichuan University, Chengdu, China

**Keywords:** Cyclosporine, Breastfeeding, Nephrotic syndrome

## Abstract

**Background:**

Cyclosporine is widely used for immunosuppressive treatment of various systematic and local autoimmune diseases. Breastfeeding is conventionally contraindicated when treating with cyclosporine due to its excretion into breast milk, which may cause immune suppression of exposed infants and affect infants` growth. A few cases have tested cyclosporine levels in random breast milk samples and concluded the infants exposed to safe cyclosporine levels during breastfeeding. Since infants do not maintain a fixed feeding schedule, we monitored cyclosporine levels in breast milk at different times of the day to assess the safety of breast milk for infants throughout the day.

**Case presentation:**

A 32-year-old dichorionic twin-pregnancy woman had nephrotic syndrome with renal biopsy confirmed type V lupus nephritis for over five years. She was treated only with prednisone 10 mg a day before pregnancy and during early pregnancy. Cyclosporine was added in her regimen from 22 weeks gestation and was adjusted to 225 mg a day from 28 weeks gestation. After parturition, she partially breastfed her twin infants while being treated with cyclosporine 3 mg/kg a day as well as prednisone and hydroxychloroquine sulfate. The cyclosporine level in maternal blood was determined, and several breast milk samples were collected for consecutive 48 h beginning on the ninth day after parturition. The concentration of cyclosporine in breast milk was measured and ranged from 0.443 to 5.307 mcg/L. Both infants grew and developed normally at the three-month follow-up, with no adverse effects observed. The study was conducted at West China Second University Hospital of Sichuan University, started in September 2021, with the consent of the participant and the approval of the ethics committee.

**Conclusion:**

In this case, cyclosporine levels in breast milk were low at all times of the day. The growth and development of both infants were normal at three months postpartum. Thus, breastfeeding may still be an option for mothers with nephrotic syndrome who are treated with cyclosporine.

**Supplementary Information:**

The online version contains supplementary material available at 10.1186/s13006-022-00514-4.

## Background

Cyclosporine, a well-known immunosuppressant, is widely used to treat various systematic and local autoimmune diseases, including severe rheumatoid arthritis, psoriasis, nephrotic syndrome (NS), severe atopic dermatitis, and uveitis, especially when conventional therapy fails [1]. Cyclosporine is permitted and regarded as safe for use in autoimmune disease patients during pregnancy [2, 3]. Breastfeeding is traditionally advised to be avoided when patients are treated with cyclosporine postpartum. This is because cyclosporine can be excreted into breast milk and be concentrated in it, which may cause infants` immune suppression and have unknown effects on growth or associated carcinogenesis to exposed infants [4–7]. Considering numerous advantages of breastfeeding, cases of successful breastfeeding by cyclosporine-treated mothers with organ transplantation, ulcerative colitis, lupus nephritis, and psoriasis have been reported in the literature in recent decades [8–12]. According to the reports, infants were exposed to very low amounts of cyclosporine via breast milk. Almost all infants had undetectable blood concentrations of cyclosporine, although one reached therapeutic blood levels [13].

However, infants do not follow a fixed feeding schedule. A random milk level of cyclosporine or an average milk level of cyclosporine for a day cannot accurately represent the level that infants consume at feeding time. Thus, data on cyclosporine concentrations in breast milk at various times of the day are required to assess the safety of breastfeeding. We present cyclosporine levels in breast milk for consecutive 48 h, in a woman with nephrotic syndrome treated with oral cyclosporine who breastfed in the postpartum period.

## Case presentation

A 32-year-old dichorionic twin-pregnancy Chinese gravida 1 para 1 had NS with renal biopsy confirmed type V lupus nephritis for over five years. She was treated with prednisone 10 mg a day before pregnancy, and NS was well controlled. She became pregnant in consultation with her follow-up nephrologist and continued to take prednisone 10 mg per day. At 22 weeks gestation, she was referred to her nephrologist because of severe proteinuria (24 h urinary protein quantity 3.041–4.952 g), and her medication regimen was adjusted to prednisone 30 mg per day as well as cyclosporine 1 mg/kg (75 mg in total) twice a day and hydroxychloroquine sulfate 200 mg per day. Her cyclosporine dose was increased to 125 mg in the morning and 100 mg at night from 28 weeks gestation, and this regimen was then maintained. At 36 weeks gestation, she received a Cesarean section, and her twin infants were born, weighing 2.4 and 2.5 kg, respectively. Both infants were male with Apgar scores of 10 at 1, 5, and 10 min.

**Fig. 1:**
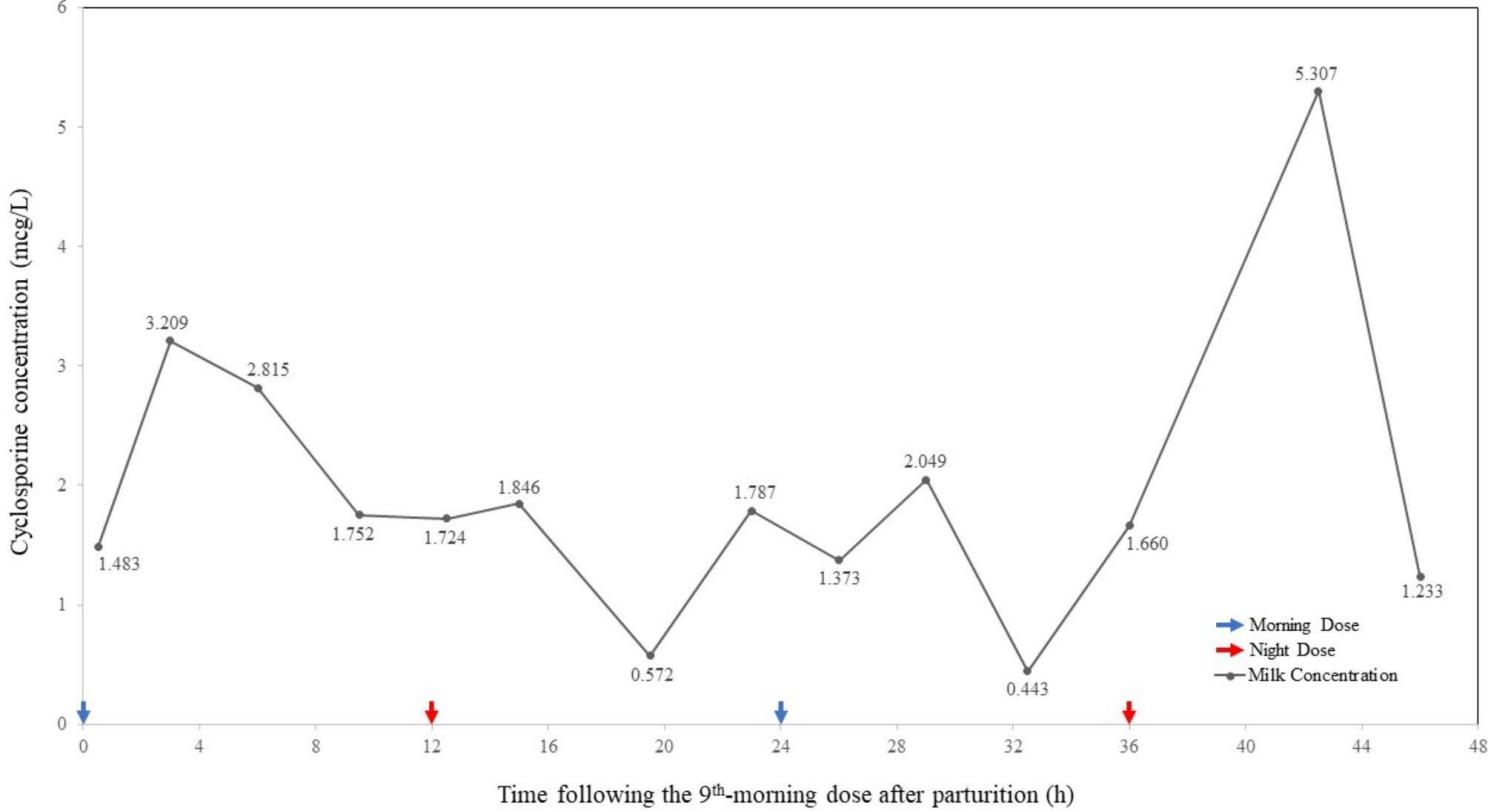
Cyclosporine concentrations in breast milk. Hindmilk samples were collected consecutively for 48 h following the 9th -morning dose of 125 mg (daily dose: 225 mg) postpartum. The drug level in the maternal blood 8th day after parturition was below the detection limit (< 30 mcg/L)

After parturition, the mother continued to take cyclosporine (3 mg/kg or 225 mg per day in two doses, 125 mg in the morning and 100 mg at night), hydroxychloroquine sulfate, and prednisone. Each medicine was to be taken at a specific time every day. Cyclosporine levels in maternal blood ranged from undetectable (< 30 mcg/L) to 43.1 mcg/L. The infants were initially given formula milk until the cyclosporine level of a milk sample two hours after a dose was gained 7 days after delivery (1.7 mcg/L). The milk cyclosporine level was initially undetectable (< 15.625 mcg/L). Thus, the laboratory method was optimized, which is described in an additional file [see Additional file 1], to achieve the exact concentration. The infants were then 70–80% breastfed since the 7th day after birth. In order to evaluate the safety of feeding on breast milk at different times of the day, hindmilk was collected every 2 to 4 h (except sleeping time) for consecutive 48 h starting from the 9th day after parturition. The mother was instructed to hand-express breast milk of the first five min each time to excrete foremilk and collect subsequent breast milk into a disposable bottle. A 2 mL breast milk sample was then extracted and stored in a sampling tube for later testing. Figure 1 depicts cyclosporine levels in breast milk. Infant blood levels were not measured since infants’ intake was low throughout the day. The mother continued to breastfeed for several months after that, and she did not receive any additional medications. The mother`s NS symptoms were alleviated. At one month, the twin infants weighed 3.9 and 4.2 kg, respectively. No adverse effects were detected in the two infants for the first three months after birth. The research was approved by the Ethics Committee for Research in Human Beings of West China Second University Hospital of Sichuan University. The mother provided written informed consent for participation in the study and publication of her and her infants` case history.

## Discussion and conclusions

Cyclosporine is a crucial agent for treating nephrotic syndrome and is safe to use in pregnant women. However, mothers taking this drug are generally advised to avoid breastfeeding because cyclosporine may pass and enrich into breast milk, which may impair renal and liver function of breastfed infants, increase their susceptibility to hypertension, hypertrichosis, gingival hypertrophy, and cancer, and lead to infants` immune suppression [14, 15]. This traditional viewpoint has gradually changed as several cases of successful breastfeeding have been reported. Thiru et al. reported an infant whose mother took cyclosporine 3 mg/kg twice daily and breastfed was healthy at two years of age [16]. Nyberg et al. reported seven infants breastfed during maternal cyclosporine grew normally [17]. The National Transplantation Pregnancy Registry reported data on mothers who breastfed their infants following organ transplantation. From 1991 to 2013, a total of 43 mothers on treatment with cyclosporine had breastfed 55 infants with no apparent adverse effects in infants [7]. Cyclosporine is then considered compatible with breastfeeding by the European League Against Rheumatism if the infants do not have conditions that preclude it [2]. The decision on drug therapy during lactation should be based on an agreement between the internist, the obstetrician, the patient, and other healthcare providers. The American College of Rheumatology indicates using cyclosporine during breastfeeding is conditionally recommended since its amount transferred into breast milk seems low [18]. Although most of published cases and series of cyclosporine-treated mothers measured cyclosporine levels in breast milk, seldom of them monitored breast milk cyclosporine concentration consecutively [13]. Osadchy et al. reported cyclosporine concentration of 46 mcg/L in one milk sample two hours after a morning dose in a post renal transplantation mother taking 2.1 mg/kg daily (175 mg in total) [8]. Mazzuoccolo et al. tested milk levels of 128–364 mcg/L in a mother with plaque psoriasis taking cyclosporine 200 mg daily [12]. Milk samples were collected before morning doses on days 10 and 40, and two hours after morning doses on days 30 and 50 postpartum. It remains unclear whether infants are exposed to safe doses anytime throughout the day when they are breastfed, especially when infants` feeding time is close to when mothers take medicine. Consecutive milk level monitoring is thus essential. In our study, the mother`s milk was safe to the infants due to the low cyclosporine levels at all times of the day (0.443–5.307 mcg/L), indicating mothers with nephrotic syndrome also have the chances to sustain breastfeeding on their infants safely like mothers received renal transplantation [17]. It was also noticed that milk cyclosporine levels in our study were much lower than that in the previous studies. It can be partially attributed to the great variability of cyclosporine metabolism among individuals and the low blood cyclosporine concentration of the mother in our study. Since our study reported the first case of mother with NS taking cyclosporine and breastfeeding, more studies on mothers with NS taking cyclosporine are worth carrying out to determine whether the disease plays a role in the metabolism of cyclosporine and causes the low milk cyclosporine levels.

During clinical practice, obstetricians and parents are eager to know the safety of cyclosporine in lactation. They are also concerned if using other medications during lactation, including other immuno-suppressants, may elevate milk cyclosporine levels. Since consecutive cyclosporine levels are measured in breast milk, general concentrations of cyclosporine intake can be valuable references to optimize the decision-making process.

Existing limited literature suggested that cyclosporine excreted into breast milk had almost undetectable effects on exposed infants, with no adverse effects reported except one infant reached “therapeutic concentration”. Moretti et al. reported one infant had blood levels of 117 and 131 mcg/L on two occasions while the peak concentration in its mother`s milk was 521 mcg/L [5]. Thus, an infant blood test can be done if the milk level is relatively high. Long-term monitoring of those children, including their vaccination status, is still necessary [14].

Although the mother in our study did not exclusively breastfeed her infants due to insufficient breast milk for the twins, concentrations of cyclosporine in breast milk were consecutively monitored and were found to be low. It seemed that the absorption, distribution, metabolism, and excretion of cyclosporine did not significantly affect cyclosporine levels in breast milk throughout the day. The growth of the two babies was normal. The maternal blood level of cyclosporine was not consecutively monitored because it was undetectable (< 30 mcg/L) on the 8th day after parturition. Thus, we did not analyze the milk blood ratio and its peak. Maternal symptoms of NS were relieved, even though her blood levels seemed under normal therapeutic concentration.

Breastfeeding should not be discouraged when mothers are on cyclosporine treatment and decide on their infants’ feeding patterns. Levels of cyclosporine excreted into breast milk during breastfeeding should be monitored if possible. When maternal blood and milk cyclosporine levels were closely and regularly monitored, breastfeeding might still be a good option. However, mothers should be fully informed about the potential risks to their infants, including the increased risks associated with live vaccines. Mothers who choose to breastfeed their infants should observe the breastfed infants for signs of infection, vomiting and poor feeding. Infants` full blood count, renal and liver function, and blood cyclosporine level should be measured if any unusual conditions are noted in the breastfed infants.

## Electronic supplementary material

Below is the link to the electronic supplementary material.

Supplementary Material 1

## Data Availability

Not applicable.

## List of abbreviations

NS: Nephrotic syndrome.
FDA: Food and Drug Administration.
