# Supplementary material for: Breastfeeding by a mother taking cyclosporine for nephrotic syndrome
Source: Int Breastfeed J. 2022 Oct 17;17:72. doi: 10.1186/s13006-022-00514-4 (PMC9578242; doi:10.1186/s13006-022-00514-4)
Supplement: Supplementary file 1 — Supplementary Material 1 [file 13006_2022_514_MOESM1_ESM.pdf]

Additional file 1. Laboratory Method.

We applied a Waters ACQUITY I-Class UPLC coupled with an Xevo TQ-S MS (Milford, MA, USA) for analysis.

Chromatographic separation was carried out using a Waters Acquity UPLC BEH C18 column (1.7 um, 50mm*2.1mm), which kept 40℃ during analysis. The mobile phase consisted of 2 mmol/L NH4Ac with 0.1% formic acid in water(A) and 2 mmol/L NH4Ac with 0.1% formic acid in methanol(B). The flow rate was 0.5mL/min. The gradient elution method was set as follows: initiation to 1 min with 50% A; 1.0-2.5 min with 50%-5% A; 2.5-3.0min with 5%-50% A; 3.0-3.5min 50% A. Sample manager was maintained at 8℃ with an aliquot of 10μl of the treated sample injected into system.

For the MS parameters, multiple reaction monitoring (MRM) mode was used with electrospray ionization source in positive mode. The capillary voltage was set at 3.0 KV. The desolvation temperature was set at 400℃ and the desolvation gas flow was set at 800 L/Hour. The gas flow of cone and nebulizer were set at 150 L/Hour and 7.0 Bar, respectively. The selected compound parameters of MRM were listed in Table 1.

Table 1 Selected compound parameters of MRM

Analyte	Q1	Q3	Cone(V)	Collision(eV)	Retention Time(min)
CSA	1219.9	1202.9	25	20	1.44
CSA-d_4	1224.1	1207.1	20	16	1.44

We then used the MassLynx software for the system control and the TargetLynx program was used for the quantification process. The area of the detected peaks, 1/X weighting and linear squares were used to create a standard curve.

The standard with a concentration of 2000μl/L of CSA was diluted to 1.95μl/L by the method of double dilution to conduct a standard curve solution. Then we added 20μl standard solution into 80μl milk blank base and mixed with 20μl internal standard and 200μl pretreatment solution. We centrifugated the breast milk samples at 12,000 rpm for 5 minutes to remove lipids. Then, 100μl of the underlying liquid was taken and mixed with 20μl internal standard and 200μl pretreatment solution. The pretreatment solution was called ISD Sample Pretreatment which was made by Roche. The reagent is content with zinc sulfate solution in methanol and ethylene glycol.

The mixed samples were vortexed at 1800 rpm for 5 minutes, and then centrifuged at 12000 rpm for 5 minutes. Finally, the supernatant was taken for LC-MS/MS analysis.
